# Timely intervention in HMG-CoA Lyase deficiency: The role of newborn screening, metabolic management, and genomic sequencing

**DOI:** 10.1016/j.ymgmr.2025.101278

**Published:** 2025-11-14

**Authors:** Iskren Menkovic, Neelam Makhijani, Ludmila Francescatto, Surekha Pendyal, Christine Stanley, Sarah P. Young, Dwight D. Koeberl, Dmitriy Niyazov, Ashlee R. Stiles

**Affiliations:** a Department of Pathology and Laboratory Medicine, Children's National Hospital; George Washington University, Washington, DC, USA; b Division of Medical Genetics, Department of Pediatrics, Duke University Medical Center, Durham, NC, USA; c Department of Pathology, Duke University Health System, Durham, NC, USA; d Variantyx, Inc. 1671 Worcester Road, Suite 400, Framingham, MA, USA; e Biochemical Genetics Laboratory, Duke University Health System, Durham, NC, USA

**Keywords:** Newborn screening, 3-hydroxy-3-methylglutaryl-CoA Lyase deficiency, Urine organic acids, Elevated Glycine, Genome sequencing, RNA expression analysis

## Abstract

3-hydroxy-3-methylglutaryl-CoA (HMG-CoA) lyase deficiency is a rare autosomal recessive metabolic disease caused by variants in the HMGCL gene leading to an impairment in leucine catabolism and ketone synthesis. In the United States, HMG-CoA lyase deficiency is listed on the recommended uniform screening panel as a core condition for newborn screening. A positive newborn screen will typically show an elevation of C5-hydroxylated species on the acylcarnitine profile using a dried-blood spot collected between 24 and 48 h of life. Initial follow-up testing generally includes a plasma acylcarnitine profile and a urine organic acid profile. Clinically, this metabolic alteration can lead to severe metabolic decompensation, presenting as hypoketotic hypoglycemia and, when left untreated, potential long-term neurological impairments. This report highlights the case of a 38-day-old male with an initial abnormal newborn screen. Follow-up testing showed moderate elevations of C5-hydroxylated and C6-dicarboxylated species on the plasma acylcarnitine profile and marked elevations of 3-hydroxy-3-methylglutaric acid, 3-methylglutaconic acid, 3-methylglutaric and 3-hydroxyisovaleric acid detected by urine organic acid analysis. These findings were consistent with a biochemical diagnosis of HMG-CoA lyase deficiency. Confirmatory molecular testing included targeted *HMGCL* sequencing including deletion/duplication analysis; the results of which were negative. Genome sequencing was then requested which identified a deep intronic complex variant of unknown significance within intron 1 of *HGMCL*. RNA sequencing studies were sent as follow-up which revealed that the level of expression of the HMGCL gene was negligible in comparison with tissue-matched controls, thus confirming the biochemical diagnosis of HMG-CoA lyase deficiency.

## 1 Introduction

3-Hydroxy-3-methylglutaryl coenzyme A (HMG-CoA) lyase deficiency (OMIM 246450) is a rare disorder of ketone synthesis secondary to a defect in leucine metabolism. Biallelic pathogenic variants in *HMGCL* results in enzymatic deficiency in the conversion of HMG-CoA into acetoacetate and acetyl-CoA. The clinical presentation typically manifests during periods of fasting or illness and includes acute metabolic decompensation, metabolic acidosis, hyperammonemia, and hypoketotic hypoglycemia. Most patients with the condition present before 1 year of age, but cases with a later onset of the condition have also been described [[1], [2], [3], [4], [5], [6]]. Although some of the acute complications are secondary to the hypoketotic hypoglycemia typically observed in patients with HMG-CoA lyase deficiency [7], recent evidence from studies on animal models seems to suggest that a toxic build-up of 3-hydroxy-3-methylglutaric acid may have adverse long-term effects on the brain homeostasis [8].

This disorder can be screened for in the newborn period by measuring 3-hydroxyisovaleryl-carnitine (C5-OH) *via* tandem mass spectrometry [9,10]. Elevated C5-OH in the newborn screen prompts confirmatory testing with urine organic acid and plasma acylcarnitine analysis [11]. Elevations of 3-hydroxyisovaleric acid, 3-methylglutaconic acid, and 3-hydroxy-3-methylglutaric acid in the urine organic acid profile and elevated C5-OH and C6-dicarboxylic (C6-DC) acylcarnitine species in plasma are highly suggestive of HMG-CoA lyase deficiency [8,12]. Other biochemical abnormalities in the setting of acute crisis may include elevated concentrations of aspartate aminotransferase (AST) and alanine aminotransferase (ALT) in addition to high lactate and ammonia levels [13]. Although biochemical testing can theoretically be sufficient to diagnose HMG-CoA lyase deficiency due to the characteristic and unique profiles observed on urine organic and plasma acylcarnitine testing, it has become common practice for providers to order molecular testing to confirm the biochemical findings. These data can also be helpful when counseling families and providing a risk assessment in the event of future pregnancies.

This manuscript describes a newborn with a biochemical diagnosis of HMG-CoA lyase deficiency based on abnormal findings in the urine organic acid and plasma acylcarnitine profiles after an initial abnormal newborn screen and highlights persistent elevations of plasma glycine. While the biochemical results were unequivocal, the molecular results returned inconclusively. Ultimately, RNA expression studies were utilized to characterize the deep intronic variants, which revealed decreased *HMGCL* mRNA, thereby confirming the biochemical diagnosis. This case highlights the strengths and weaknesses of various clinical tests and the necessity of a multidisciplinary approach when diagnosing rare inborn errors of metabolism.

## 2 Methods

The clinical team reviewed the patient's medical record and clinical parameters.

Duke University Health System's (DUHS) CLIA/CAP-certified Biochemical Genetics Laboratory performed the urine organic acid and plasma acylcarnitine analyses according to standard operating procedures as previously published [14]. Plasma amino acid analysis was performed by high-performance liquid chromatography cation exchange system with ninhydrin detection using the Biochrom 30 Amino Acid Analyzer (Biochrom, Cambridge, UK) according to manufacturer's protocol and standard operating procedure at DUHS Biochemical Genetics Laboratory.

Single gene testing was performed at GeneDx and at Invitae. Genome sequencing was performed at Variantyx. RNA expression analysis was performed at MNG Labs (Labcorp).

### 2.1 Clinical presentation

**Fig. 1:**
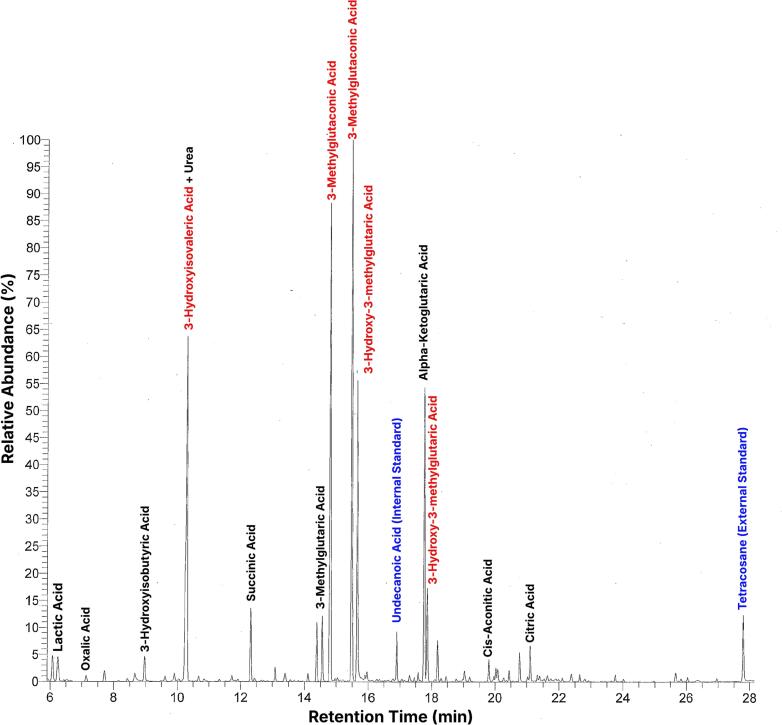
Total ion chromatogram (TIC) of urine organic acid profile. Significant peaks excreted are denoted in red, internal and external standards are in blue, and routinely observed metabolites in black.

**Fig. 2:**
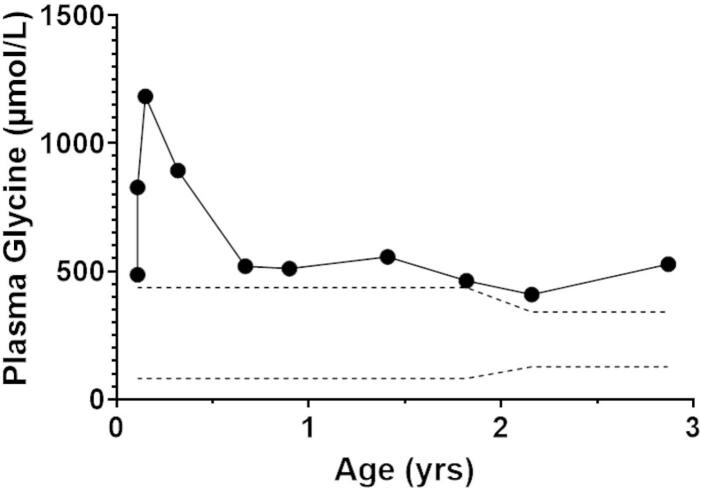
Trends in plasma glycine concentrations show persistent elevations. The upper and lower limits of the normal reference range are denoted by the dashed lines.

The proband is an adopted male who was born at 37-weeks of gestation with limited prenatal and familial history available. Initial dried-blood spot newborn screening performed on day two of life showed an elevation of the C4-DC/C5-OH isobaric pair acylcarnitine species (0.94 μM and 1.06 μM on initial and repeat analyses, reference limit <0.78 μM). Confirmatory metabolic testing collected at 35 days of life included urine organic acid analysis that revealed marked elevations of 3-hydroxy-3-methylglutaric acid, 3-methylglutaconic acid, and 3-hydroxyisovaleric acid in a pattern consistent with HMG-CoA lyase deficiency (Fig. 1). These abnormal results were urgently communicated to the ordering provider who instructed the family to report to the emergency department (ED) at the onset of any signs or symptoms characteristic of HMG-CoA lyase deficiency, such as lethargy, vomiting, poor feeding, irritability, hypotonia, unresponsiveness. These results, communicated to the family late on a Friday afternoon, coincided with the patient's presentation to the ED later that same weekend, on day of life 38, with lethargy and projectile vomiting. Laboratory evaluation revealed elevated lactic acid with normal plasma ammonia. Plasma acylcarnitine and amino acid profiles collected on day of life 39 revealed elevated C5-OH/C3-DC (0.31 μmol/L; NL < 0.07) and C6-DC-carnitine species (0.39 μmol/L; NL < 0.15), and a mild elevation of glycine which rapidly increased over the course of the admission (Fig. 2). The patient was managed with intravenous 10 % dextrose, levocarnitine, and a specialized diet including leucine-restricted formula Anamix, synthetic beta-hydroxybutyrate and Polycal. The diet was carefully adjusted to limit leucine intake and reduce fat to 30 % of total calories, aligning with treatment recommendations for HMG-CoA lyase deficiency [12]. Subsequently the patient had decreased vomiting and improved feeding. Plasma amino acids subsequently revealed a normal concentration of leucine, a mild elevation of alanine, and an elevation of glycine. Aside from two hospitalizations for gastroenteritis, the patient remains metabolically stable without hypoglycemia which can be attributed to the ongoing dietary management and biochemical monitoring of HMG-CoA lyase deficiency. Amino acid monitoring continued to show persistent mild elevations of glycine over the course of three years. Acylcarnitine monitoring continued to show a persistent elevation of C5-OH carnitine, but not C6-DC carnitine. Cognitive development has been close to age appropriate with normal motor development and mild expressive language delay which has been improving with speech therapy.

The patient has had no further hospitalization or acute complications and has had normal growth. His medical history also includes hypospadias, as well as brachycephaly requiring helmet therapy. The patient continues to thrive and is followed in metabolic clinic every 6 months for monitoring including biochemical assessments and dietary adjustments as needed.

### 2.2 Genetic testing

**Fig. 3:**
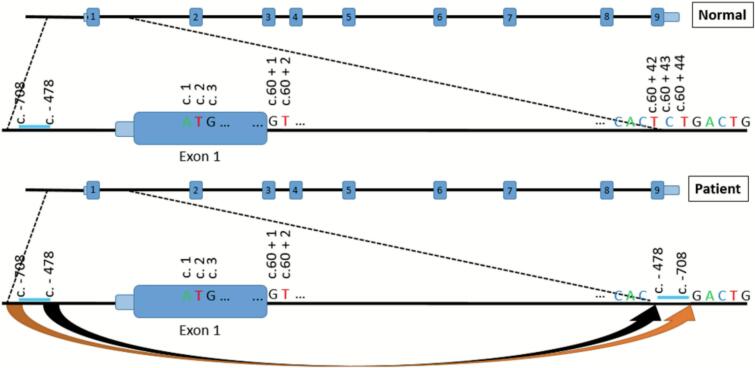
Schematic illustration of the deep intronic variant c.60 + 42_60 + 44delins-708_-478inv(p?) in *HMGCL*. This complex event involves a 3-bp deletion (c.60 + 42_60 + 44del; chr1:23825312–23,825,314) with the concomitant insertion, in reverse orientation, of a duplicated 231-bp segment (chr1:23825893–23,826,123) from upstream of *HMGCL*.

SNP microarray analysis revealed several regions of homozygosity encompassing >27.6 % of the genome which suggested a first- or second-degree familial relationship. Of note, one of the regions of homozygosity (chr1:20611319–121,339,317) included *HMGCL*. Subsequently, *HMGCL* targeted sequencing was performed independently by two different testing laboratories but failed to identify any variants of significance. Due to a biochemical diagnosis of HMG-CoA lyase deficiency and negative targeted gene testing, genome sequencing was performed as an attempt to discover any significant variants in regions not covered by targeted single gene testing, such as deep intronic and promoter regions. Genome sequencing identified a homozygous deep intronic complex variant of uncertain significance (VUS) in *HMGCL*, c.60 + 42_60 + 44delins-708_-478inv(p?) (Fig. 3). Bioinformatic tools, or computational predictions, suggested that this variant affects *HMGCL* mRNA splicing (https://spliceailookup.broadinstitute.org); however, the variant lacked sufficient evidence for pathogenicity and was classified as a VUS per ACMG guidelines (Richard et al., 2015). To better understand the impact of this variant on the expression of this gene, RNA sequencing was performed and showed an overall expression level of *HMGCL* to be negligible compared with tissue-matched controls (*Z*-score = −22.7; reference range: −2.0 to 2.0).

According to recent recommendations for clinical interpretation of variants found in non-coding regions of the genome, and from the ClinGen SVI Splicing Subgroup, PVS1_Strength code may be applied for variants with experimental evidence showing aberrant RNA transcript(s) with loss of function [15]. Therefore, this variant may be reclassified as likely pathogenic.

## 3 Discussion

This case report highlights the importance of NBS and the necessity to have timely and efficient communication between the healthcare providers and the laboratory team. The results of urine organic acid analysis supported the abnormalities detected in the newborn screening process and were consistent with a diagnosis of HMG-CoA lyase deficiency. These laboratory results were critical in allowing our team to promptly contact the provider, and subsequently the family, with clear instructions and a protocol to follow, should symptoms manifest.

HMG-CoA lyase is essential for ketogenesis and the final step in leucine oxidation. Deficiency in this enzyme places patients at risk for metabolic decompensation, often presenting as hypoketotic hypoglycemia, which can progress to coma or death. Long-term complications include intellectual disability, epilepsy, and spasticity. Treatment involves protein restriction to limit leucine intake, reduced dietary fat due to impaired fatty acid oxidation, and avoidance of fasting [12].

Our patient was managed with intravenous (IV) 10 % dextrose at 1.5 times maintenance (8 mg glucose/kg/min) and elimination of dietary protein at the time of presentation with symptoms indicating metabolic decompensation. Subsequently he was managed with levocarnitine supplementation (200 mg/day orally and 450 mg/day from metabolic formula), and dietary therapy. At diet initiation, on the metabolic dietitian recommended a combination of IVA Anamix Early Years (leucine-free), Gerber Good Start Gentle (to meet daily leucine requirements), and Polycal (carbohydrate-only supplement to reduce fat intake to ∼30 %). At 3 months of age, synthetic ketones (BHB, KetoForce) were introduced at ∼400 mg/kg/day, with parents reporting positive clinical effects. His diet was tailored to meet his daily recommended intake for protein, leucine, and calories, and was continuously adjusted based on growth, appetite, and plasma leucine levels. For example, at two years of age a metabolic formula containing Valex 1, 1 % fat cow's milk, and Polycal provided 540 Kcal, 9.75 g protein, and 250 mg leucine, and a vegan diet provided 18 g/day with a total protein intake of 28 g/day (1.5 g/kg/day). Following initiation, the patient showed improved feeding and reduced vomiting.

The patient experienced two subsequent hospitalizations for gastroenteritis, during which emergency protocols (IV dextrose, carnitine supplementation, and “sick day” formula) were implemented promptly. Sick-day formula consisted of a leucine-free formula with supplemental carbohydrates to provide 540 Kcal, 8.25 g protein, 125 mg leucine, and 92.97 g carbohydrates with dietary protein held for 24 to 48 h, until symptoms improved. He remained metabolically stable, without acidosis or hypoglycemia. At 12 months of age, persistent mild elevations of plasma glycine prompted a switch from IVA Anamix to Valex I. Although we have not found other reports in the literature of elevated glycine in HMG-CoA lyase deficiency, it is well-documented that plasma glycine is elevated in organic acidemias [16], possibly due to suppression of the glycine cleavage complex. Furthermore, our laboratory has diagnosed two other siblings with HMG-CoA lyase deficiency, both of whom had hyperglycinemia (unpublished data). Of note, the persistent elevation of C5-OH carnitine, but not C6-DC carnitine, noted in our patient has also been observed in other cases of treated HMG-CoA lyase deficiency (unpublished data).

This case also illustrates the strengths and limitations of genetic testing in the diagnostic evaluation of rare metabolic disorders [17]. The patient's metabolic profile confirmed HMG-CoA lyase deficiency caused by variants in *HMGCL*. While targeted gene sequencing was uninformative in our patient—failing to detect a pathogenic variant despite a strong clinical and biochemical phenotype—genome sequencing identified a deep intronic VUS. Intronic variants, particularly those outside canonical splice sites, often evade interpretation due to limited predictive power of *in silico* tools and lack of functional evidence. To address this uncertainty, RNA expression analysis was performed, which revealed aberrant splicing consistent with loss of function. This functional evidence enabled the reclassification of the variant from VUS to likely pathogenic, thereby confirming the biochemical diagnosis. This approach aligns with emerging best practices in genomic medicine, where reflex RNA sequencing is increasingly used to resolve ambiguous findings from exome or genome sequencing—especially in cases involving suspected splicing defects [18]. Recent studies have shown that integrating RNA analysis into the diagnostic workflow can significantly improve variant interpretation. For example, in one cohort, 50 % of intronic or splice-region VUS were reclassified as likely pathogenic based on RNA evidence [19]. This case underscores the value of comprehensive genomic approaches, particularly when initial testing is inconclusive, and highlights the importance of genome sequencing in identifying variants in noncoding regions and the critical role of RNA-based functional assays in establishing pathogenicity for non-coding variants.

Importantly, this case underscores the life-saving potential of newborn screening. Early identification of HMG-CoA lyase deficiency through NBS enabled pre-symptomatic diagnosis and timely intervention, likely preventing a severe metabolic decompensation. Studies have shown that early detection through NBS significantly reduces the risk of neurological damage, coma, and death associated with this disorder [9,10,20]. The inclusion of HMG-CoA lyase deficiency in NBS panels reflects its treatability and the substantial benefit of early intervention [11]. This case exemplifies how timely communication of critical lab results can directly impact clinical outcomes and highlights the importance of robust follow-up systems to ensure families receive and understand critical health information.

## 4 Conclusion

This case underscores the life-saving potential of NBS and the critical importance of timely communication of abnormal confirmatory testing results between clinical teams and laboratories in the early diagnosis and management of HMG-CoA lyase deficiency. It also highlights the evolving role of comprehensive genomic testing, particularly RNA expression analysis, in resolving uncertain genetic findings and guiding targeted interventions. Together, these tools enabled early treatment, prevented metabolic crises, and supported favorable clinical outcomes in an otherwise high-risk patient.

## CRediT authorship contribution statement

**Iskren Menkovic:** Methodology, Formal analysis, Writing – original draft. **Neelam Makhijani:** Investigation, Writing – original draft. **Ludmila Francescatto:** Visualization, Investigation, Formal analysis, Data curation. **Surekha Pendyal:** Investigation, Writing – original draft. **Christine Stanley:** Resources, Formal analysis, Data curation. **Sarah P. Young:** Formal analysis, Data curation, Writing – review & editing. **Dwight D. Koeberl:** Visualization, Supervision, Investigation, Conceptualization, Writing – review & editing. **Dmitriy Niyazov:** Supervision, Investigation, Conceptualization, Writing – review & editing. **Ashlee R. Stiles:** Visualization, Supervision, Methodology, Conceptualization, Writing – review & editing.

## Declaration of competing interest

None.

## Data Availability

Data will be made available on request.
